# pH Modulated Formation of Complexes with Various Stoichiometry between Polymer Network and Fe(III) in Thermosensitive Gels Modified with Gallic Acid

**DOI:** 10.3390/gels9060447

**Published:** 2023-05-29

**Authors:** Klaudia Kaniewska, Patrycja Kościelniak, Marcin Karbarz

**Affiliations:** Faculty of Chemistry, Biological and Chemical Research Center, University of Warsaw, 1 Pasteura Str., PL-02-093 Warsaw, Poland

**Keywords:** gallic acid, thermosensitive gel, pH-sensitive gel, iron complex, volume phase transition

## Abstract

Thermoresponsive gels based on *N*-isopropylacrylamide functionalized with amino groups were modified with gallic acid, with gallate (3,4,5-trihydroxybenzoic) groups being introduced into the polymer network. We investigated how the properties of these gels were affected at varying pH, by the formation of complexes between the polymer network of the gels and Fe^3+^ ions (which form stable complexes with gallic acid, exhibiting 1:1, 1:2, or 1:3 stoichiometry, depending on pH). The formation of complexes with varying stoichiometry within the gel was confirmed using UV-Vis spectroscopy, and the influence of such complexes on swelling behavior and volume phase transition temperature were investigated. In the appropriate temperature range, complex stoichiometry was found to strongly affect the swelling state. Changes in the pore structure and mechanical properties of the gel caused by the formation of complexes with varying stoichiometry were investigated using scanning electron microscopy and rheological measurements, respectively. The volume changes exhibited by p(NIPA-5%APMA)-Gal-Fe gel were found to be greatest at close to human body temperature (~38 °C). Modification of thermoresponsive pNIPA gel with gallic acid opens new opportunities for the development of pH- and thermosensitive gel materials.

## 1. Introduction

Gallic acid (3,4,5-trihydroxybenzoic acid, GA) is an interesting compound present in many areas of life—for instance, it is commonly used in the ink, dye, food, and pharmaceutical industries [1]. GA is a phenolic compound (a secondary polyphenolic metabolite) naturally most abundant in fruits, nuts, and tea leaves. Salts of gallic acid are known as gallates. Gallic acid possesses valuable medical properties, such as antioxidant, nephrotoxic, and anti-cancer characteristics [2,3,4]. Among other applications, this compound was commonly used as an ingredient in medieval writing inks, due to the formation of color complexes [5]. Gallic acid can form strong complexes with metal ions, of which the color and, therefore, the stoichiometry, depends on the pH. With Fe^3+^, pale yellow, brownish, and violet complexes are known, with a stoichiometry of 1:1, 1:2, and 1:3, respectively [6,7]. The stability constant of Fe^3+^ with gallic acid complexes is logβ = 25.8 [8].

The interesting properties of gallic acid have promoted the compound as an attractive candidate for introduction into polymer networks to obtain new multifunctional materials [9,10]. Guo and co-workers grafted gallic acid to hyaluronic acid chelate iron(III) and combined it with Ce6, applying this system in melanoma synergistic therapy [11]. Kim et al. presented an interpenetrating polymer network consisting of chitosan and poly(2-hydroxyethyl methacrylate), surface-modified with gallic acid. The hydrogel obtained possessed antioxidant properties, and could serve as a material for creating biomedical devices [12]. Another application—an antibacterial liquid bandage—was presented by Supaphol et al., with gallic acid–copper iodide nanoparticles being loaded into poly(vinyl alcohol) [13]. The combination of hydrogel properties such as softness and sorption capacity, with the properties of gallic acid such as antioxidant and anti-inflammatory qualities, make such materials an excellent candidate for wound dressing [14,15,16,17]. The combination of gallic acid with hydrogels that serve as drug delivery systems are also worth mentioning [18,19]. Gels based on gallate analogues were investigated in terms of pH effect on the mechanical properties of gels, their ability to create crosslinks in a water environment, and their self-healing properties; this was mostly PEG analogs [20,21,22].

One very interesting feature is the stimulus-responsiveness of hydrogel materials. Materials that can change volume quickly and reversibly upon environmental changes are called smart or “intelligent”. A change in volume can be triggered by an external stimulus such as temperature, pH, ionic strength, UV light, an electric field, etc. [23,24,25,26,27,28]. One of most well-investigated thermosensitive polymers is pNIPA [29,30,31]. pNIPA hydrogel has a low critical soluble temperature (LCST = 32 °C); below this, the gel is in a swollen state, whereas, above it, the gel contracts and loses water [32]. The temperature of the volume phase transition can be shifted by introducing monomers with different hydrophilicity into the pNIPA network. A more hydrophilic additive will shift the temperature to higher values, while at the same time increasing the swelling ratio. A drop in LCST temperature and decrease in swelling ratio can be changed by introducing monomers more hydrophobic than NIPA. For example, acrylic acid (AA) monomers introduced to the pNIPA network will increase the volume-phase temperature: a 7 mol% addition of AA, for instance, increased LCST to 40 °C [33], whereas the addition of 5 mol% dopamine methacrylamide to pNIPA gel decreased LCST to 28 °C [34]. Another common trigger is pH. Changes in volume are usually related to protonation/deprotonation of hydroxyl groups, and shrinking and swelling relate to osmotic pressure changes along with a change of charge on the polymer network [35]. There are limited specific systems where the pH-sensitivity depends on another mechanism, for example, on an oxidation/reduction reaction that changes the hydrophilicity of the polymer network such as in quinine-modified hydrogels [36], or as presented in this work where pH affects the stoichiometry of complexes and, therefore, alters the crosslinking points’ functionality. More demanding hydrogels are sensitive to more than one external stimulus. Such materials can be obtained, for instance, by the creation of interpenetrating polymer networks [37], a double network [38], or copolymers [39,40], etc. Stimulus-responsive materials that trigger mechanical action are particularly interesting due to their potential to act as actuators, artificial muscles, or soft robots [41,42,43,44,45,46]. The ability to form complexes between metal ions and appropriately designed polymer networks, with different stoichiometry, and its effect on swelling ratio, can be utilized to trigger volume changes by different stimuli [34,47,48].

Herein, we present a thermosensitive hydrogel based on pNIPA, modified with gallic acid, to yield a both temperature- and pH-sensitive gel. Modifying the polymer network with gallate groups permitted the use of nontoxic iron(III) as a cross-linking agent. The pivotal role of iron ions lies in creating complexes with polyphenol groups attached to the polymer chain. The stoichiometry of iron:gallate complexes depends on the environmental pH. Not only do the volume and temperature of the volume phase transition change, but so does the hydrogel color. In an optimal composition, the hydrogel is sensitive to pH due to complex ratio changes, and to temperature due to the presence of NIPA. In this work, we obtained a hydrogel that had a wide temperature window in which the volume could be changed drastically. Additionally, compared with a previous report [34], the pH and temperature triggered significantly higher volume changes.

## 2. Results and Discussion

**Figure 1:**
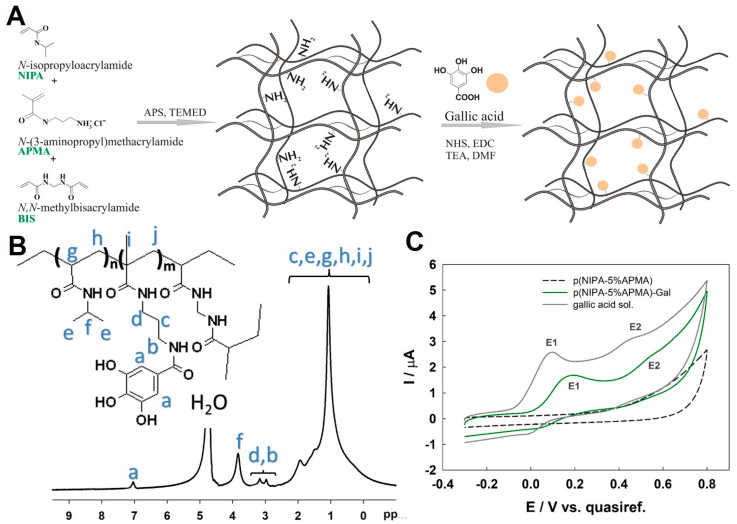
(**A**) Scheme of the synthesis of p(NIPA-APMA) gels and their modification with gallic acid to obtain p(NIPA-APMA)-Gal hydrogels. (**B**) ^1^H NMR spectra of p(NIPA-5%APMA)-Gal gel. (**C**) Cyclic voltammograms recorded for the gel before modification p(NIPA-APMA) (black dashed line), after modification with gallic acid p(NIPA-APMA)-Gal (green line), and in 0.1 mM solution of gallic (grey line) acid; scan rate 50 mV·s^−1^.

The scheme of synthesis of p(NIPA-X%APMA) gels and their modification with gallic acid (p(NIPA-X%APMA)-Gal) is presented in Figure 1A. To verify that the polymeric network was successfully modified, ^1^H NMR spectra were recorded. To this aim, the p(NIPA-5%APMA)-Gal gel sample was prepared by swelling the pieces of dry polymeric network in D_2_O. A typical gel spectrum for (NIPA-5%APMA)-Gal gel is shown in Figure 1B. Characteristic signals for NIPA (f), APMA (b and d), and gallic moiety (a), were well visible. However, no distinctive signals from the BIS could be observed, as the mole fraction of the cross-linker was kept at a low level (1%), and the signals overlapped with the much larger ones. Importantly, the area (integral) of the signal corresponding to the two aromatic protons of the gallate groups (a) were almost two times smaller than the area of the b and d signals associated with the four protons of APMA monomers. This suggested that the modification of APMA monomers in the polymer network was close to quantitative. The presence of the gallate groups in the polymeric network of the gel was also confirmed using electrochemical methods. Voltammograms obtained for p(NIPA-5%APMA) and p(NIPA-5%APMA)-Gal gels are shown in Figure 1C. The p(NIPA-5%APMA)-Gal showed an electrochemical response typical for gallic acid (grey line), whereas the hydroxyl groups were irreversibly oxidized; this was also in agreement with the literature [49,50]. Overall, the electrochemical process was a two-electron two-proton process, split into two steps (marked in Figure 1 as E1 and E2). The first peak corresponded to irreversible oxidation of the hydroxyl group to semiquinon radical form, with one proton lost (E1). The second peak related to one-electron irreversible oxidation and dehydrogenation of the semiquinone radical to quinone (E2). The observed voltametric response meant that the gallate groups were in the reduced state. The unmodified hydrogel (black dashed line) gave no faradaic response.

**Figure 2:**
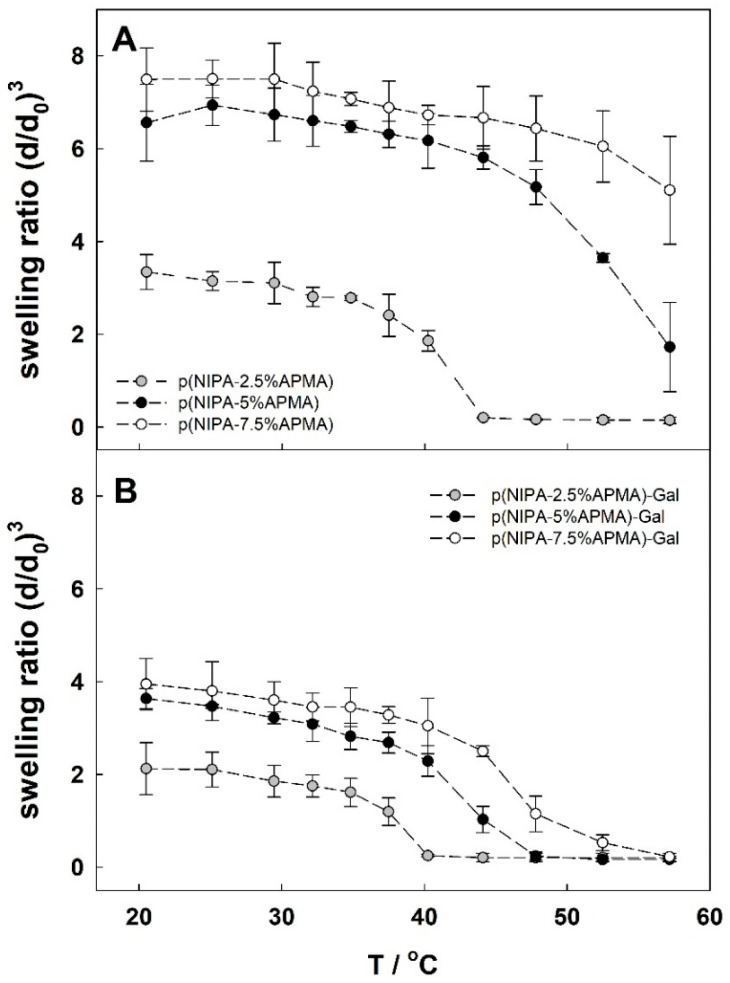
Swelling ratio changes by temperature for (**A**) unmodified hydrogels with different amounts of APMA monomer, and (**B**) the same hydrogels modified with gallic acid, in 1 mM NaNO_3_.

Next, the influence of modifying p(NIPA-x%APMA) with gallic acid on swelling behavior was investigated. Polymeric hydrogels based on poly(*N*-isopropylacrylamide) (pNIPA gels) are commonly known to be thermosensitive and exhibit a drastic swelling transition at their lower critical solution temperature (LCST) of ca. 32 °C. At temperatures below 32 °C, the gels are swollen, whereas at temperatures higher than 32 °C, the gels dehydrate to the collapsed state [32]. Figure 2A presents the swelling ratio as a function of temperature for gels containing different amounts of APMA monomer. Increasing amounts of APMA monomer in the gel network strongly increased the swelling ratio and shifted the volume phase transition temperature (VPTT) to higher values. The measurements were performed in neutral pH; in these conditions the amine groups in the polymer network were practically fully protonated (pKa of APMA is around 9 [51]). The ionized groups were very hydrophilic and created an osmotic pressure in the hydrogel, which resulted in high water absorption and shifted VPTT to higher values. For p(NIPA-2.5%APMA) and p(NIPA-5%APMA), the VPTT was ca. 41 °C and 52 °C, respectively, whereas p(NIPA-7.5%APMA) gel practically lost its thermosensitivity in the investigated temperature range. After modification of the gels with gallic acid, amino groups were converted into amide groups and both swelling ratio and temperature decreased significantly, see Figure 2B. At neutral pH, added moieties (gallate groups) were less hydrophilic than ionized amine groups. The modification of the network with gallic acid led to a drop in the osmotic pressure due to the vanishing of protonated amine groups (from APMA units) and, therefore, diminished the repulsive interaction between polymer chains. Altogether, it led to a decrease in the VPTT value compared with hydrogels unmodified with gallic acid. The temperatures shifted to ca. 38 °C, 42 °C, and 45 °C for p(NIPA-2.5%APMA)-Gal, p(NIPA-5%APMA)-Gal, p(NIPA-7.5%APMA)-Gal, respectively.

**Figure 3:**
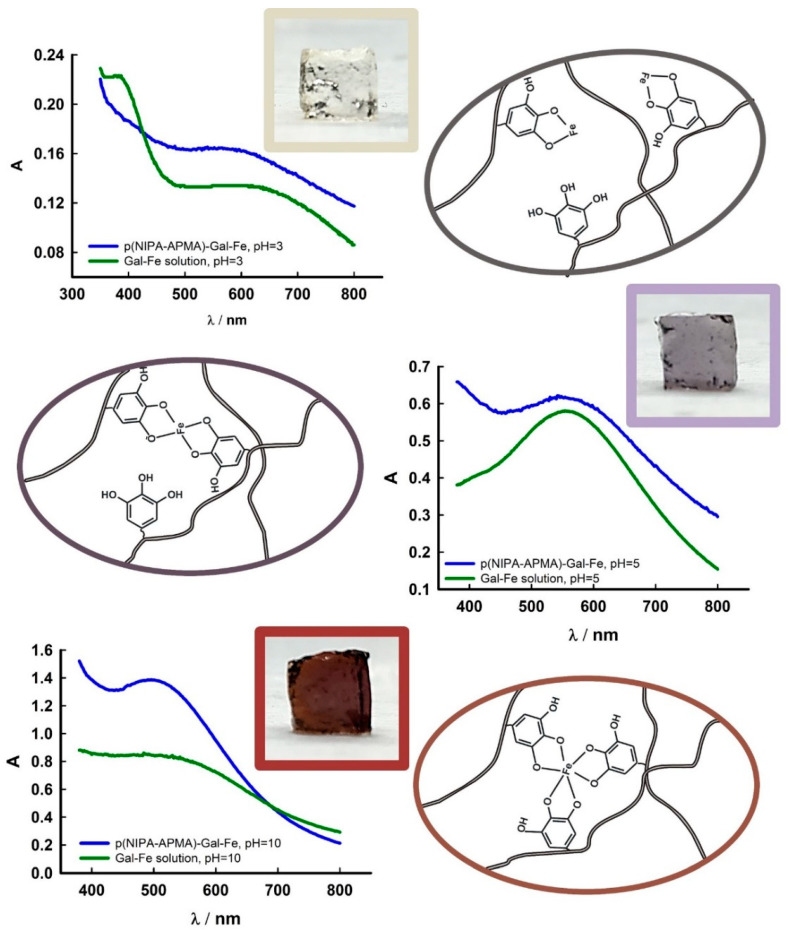
Photography of p(NIPA-5%APMA)-Gal-Fe(III) gels samples at pH 3, 5, and 10, accompanied by a scheme of formed complexes. The UV-Vis spectra of the hydrogel samples and gallic acid/Fe(III) solutions at given pH.

Next, the ability of the gallate groups attached to the polymer network to form complexes with Fe(III) ions was investigated. Gallic acid is known to form complexes with Fe(III) with stoichiometry depending on the pH of the solution, with the color of the complexes changing with complex stoichiometry [6]. Figure 3 shows photography of pNIPA-5%APMA-Gal hydrogels with Fe(III) in different pH. p(NIPA-5%APMA)-Gal-Fe gel in pH = 3 exhibited a pale yellow–grey color, and the complex gallate group-Fe(III) ion had a stoichiometry of 1:1. When the pH was changed to 5, in turn, the complex stoichiometry changed to 2:1 and the gel became violet. For pH = 10, p(NIPA-5%APMA)-Gal-Fe gel exhibited a burgundy color, which meant that the stoichiometry of the gallate-iron(III) complex changed to 3:1. The scheme of complex formation is shown in Figure 3. UV-Vis spectra were recorded for the swollen gel samples and compared with respective spectra of complexes of gallic acid and Fe(III) in aqueous solution at room temperature 21 °C. A blue shift was observed for the gels, the wavelengths for maximum of absorption being shifted to lower wavelengths compared with the solution. Moreover, the color of the gel at pH = 3 was pale, and in this case, a peak was barely visible. However, based on comparison of the spectra it was concluded that similar complexes were formed between Fe(III) and gallate groups from the polymer network in the gel and gallic acid in the solution.

**Figure 4:**
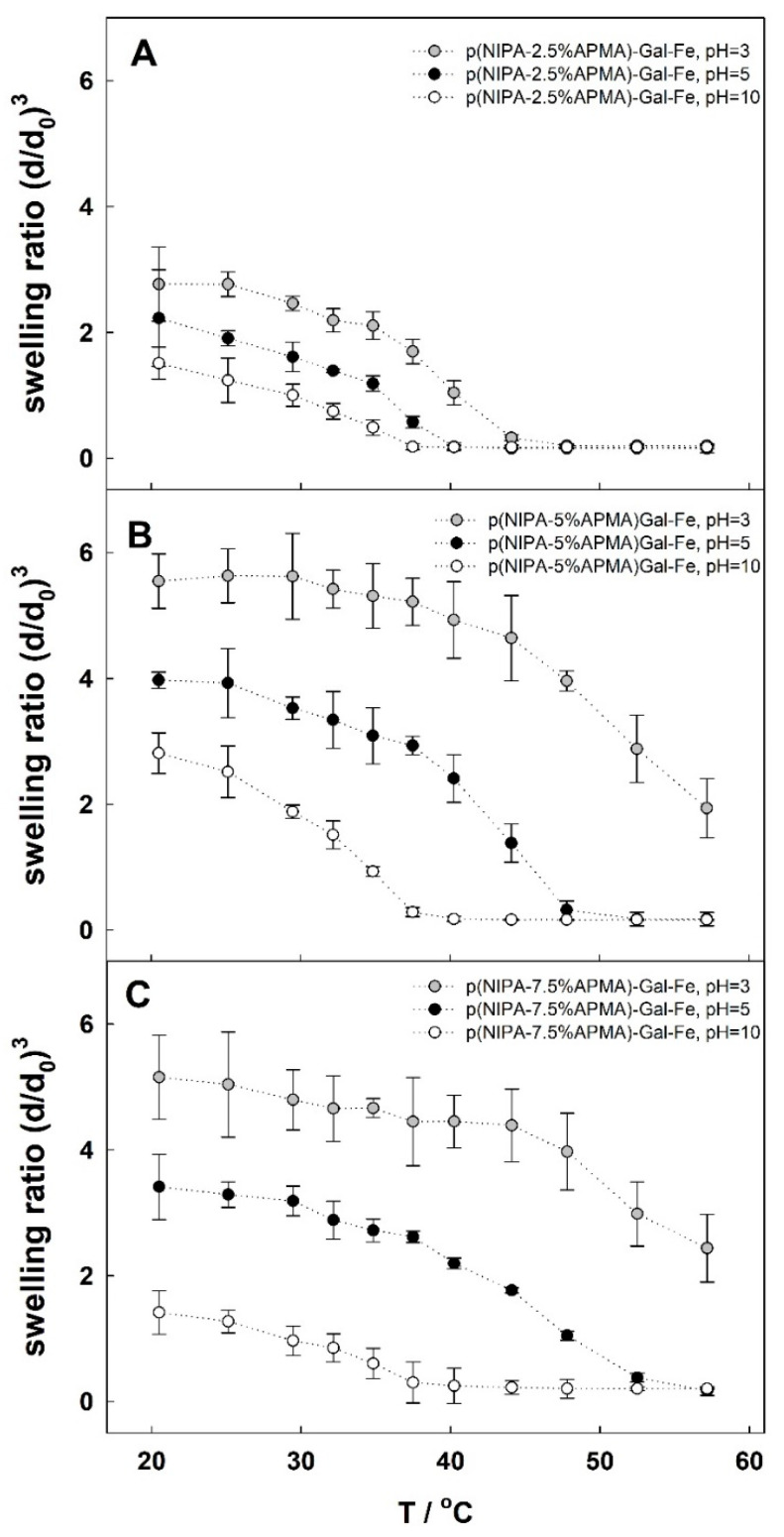
Swelling ratio changes upon temperature for hydrogels p(NIPA-X%APMA) modified with gallic acid and iron ions in different pH (in 1 mM NaNO_3_, except pH = 3) for hydrogel (**A**) p(NIPA-2.5%APMA)-Gal-Fe, (**B**) p(NIPA-5%APMA)-Gal-Fe, and (**C**) p(NIPA-7.5%APMA)-Gal-Fe.

Lastly, the effect of the Fe(III) ion complexation by the polymer network on the properties of the gels was studied. Due to the formation of Fe(III) complexes with gallate groups of different stoichiometry, depending on the pH, it was expected that the resulting material would be pH- and thermosensitive. First, the swelling behavior of p(NIPA-X%APMA)-Gal-Fe(III) gels was studied as a function of temperature at the selected pH values of 3, 5 and 10, with predominant complexes of stoichiometry of 1:1, 1:2 and 1:3, respectively. The obtained results are presented in Figure 4. It was evident that for each p(NIPA-X%APMA)-Gal-Fe(III) gel, both the swelling ratio and the VPTT decreased with increasing pH, in the order 3, 5, 10. At pH = 3, complexes with 1:1 stoichiometry predominated and no additional physical cross-links were formed. In addition, positive charges were introduced into the polymeric network, leading to an increase in the swelling ratio and VPTT compared with gel without Fe(III) (see Figure 2B). Increasing the pH to 5 resulted in the formation of complexes with a stoichiometry of 1:2, meaning that two gallate groups were involved in each complex and an additional physical crosslink point appeared (the gel shrank and turned violet). Alkalization to pH 10, in turn, led to a further decrease in the swelling ratio and shifted the VPTT to a lower value. This was explained by the creation of complexes with a stoichiometry of 3:1, meaning that Fe(III) created physical crosslink points that involved three gallate groups. Therefore, the increase in pH caused increases in the functionality of the physical crosslink points from two, to four, to six. The shift in VPTT for different complexes creates the opportunity to generate a larger pH-triggered change in volume. From this point of view, the most interesting ranges of temperature are those where the gels with 3:1 complexes shrink (after volume phase transition), while those with 2:1 and 1:1 complexes swell (before volume phase transition). These conditions allow the largest pH-dependent volume changes to be achieved. This temperature range for p(NIPA-2.5%APMA)-Gal-Fe is narrow, and the volume changes are relatively small. For p(NIPA-5%APMFAA)-Gal-Fe and p(NIPA-7.5%APmA)-Gal-Fe gels, the temperature range and volume changes are significantly wider. Once again, optimal hydrogel composition is where the widest temperature window, and the biggest volume changes, are observed. However, for p(NIPA-5%APMA)-Gal-Fe, the volume changes are largest around the temperature of the human body (~38 °C), and so this gel was selected for further study.

**Figure 5:**
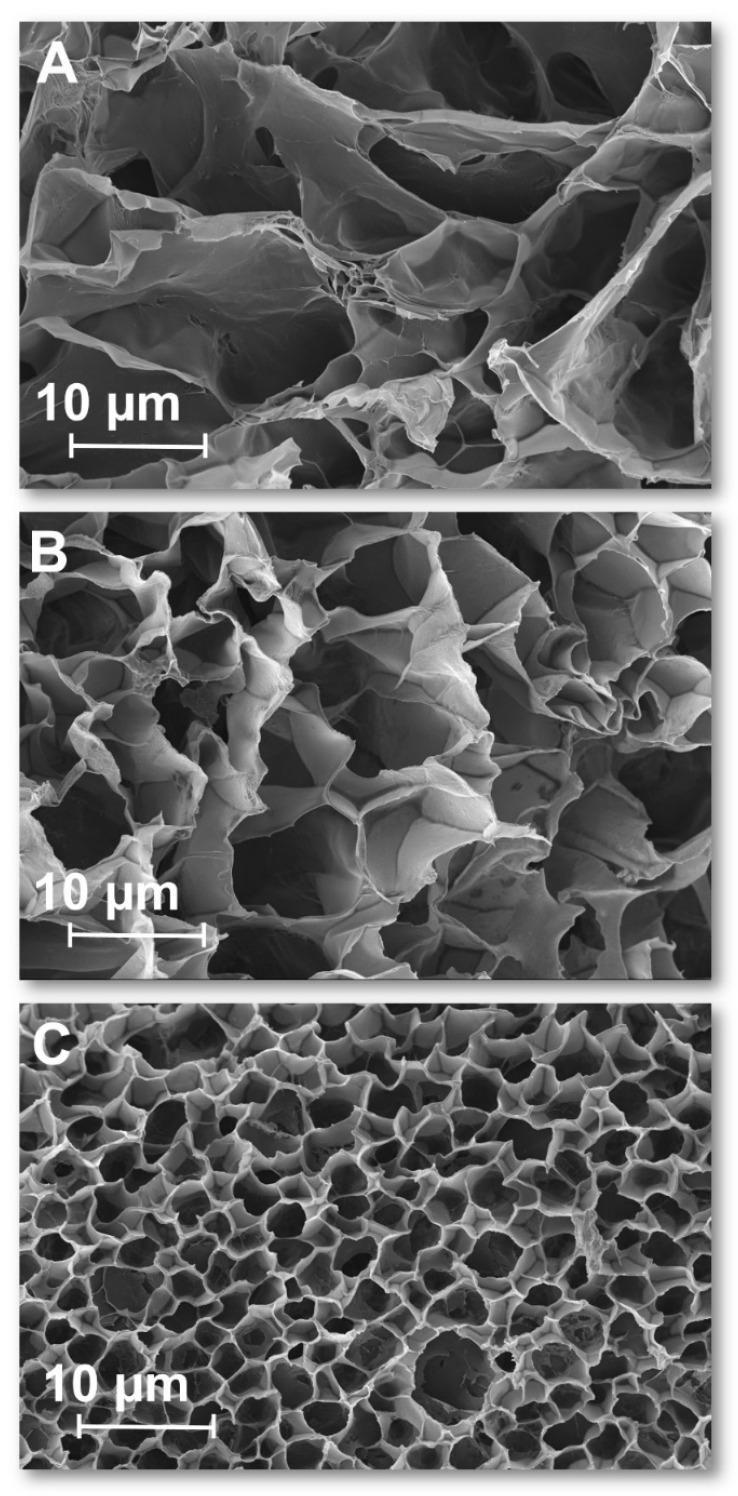
SEM images of lyophilized p(NIPA-5%APMA)-Gal-Fe hydrogel samples conditioned at various pH: (**A**) pH = 3, (**B**) pH = 5, (**C**) pH = 10.

To visualize how pH affects the hydrogel structure, the morphology of three samples of p(NIPA-5%APMA)-Gal-Fe were examined. The samples were conditioned at pH 3, 5 and 10, then lyophilized and investigated using SEM. The obtained images are presented in Figure 5. The pore structure of the gel sample was well visible. The pore size was the biggest for p(NIPA-5%APMA)-Gal-Fe conditioned at pH = 3 (Figure 5A). There were no additional cross-linking points and the gel was in an expanded state, the average pore size diameter was equaled at 21.25 ± 4.39 µm. For p(NIPA-5%APMA)-Gal-Fe hydrogel at pH = 5, the diameter of the channels decreased to an average value of 8.9 ± 1.53 µm as a result of additional cross-linking points appearing, arising from the interaction of iron ions with two gallate groups. The smallest pores were observed for the gel conditioned at pH = 10 (Figure 5B); their average size was 3.48 ± 1.15 µm, as expected for a gel where three gallate groups attached to a polymer chain interacted with one iron(III) ion. These results were in good agreement with data obtained from swelling behavior experiments, see Figure 4B.

**Figure 6:**
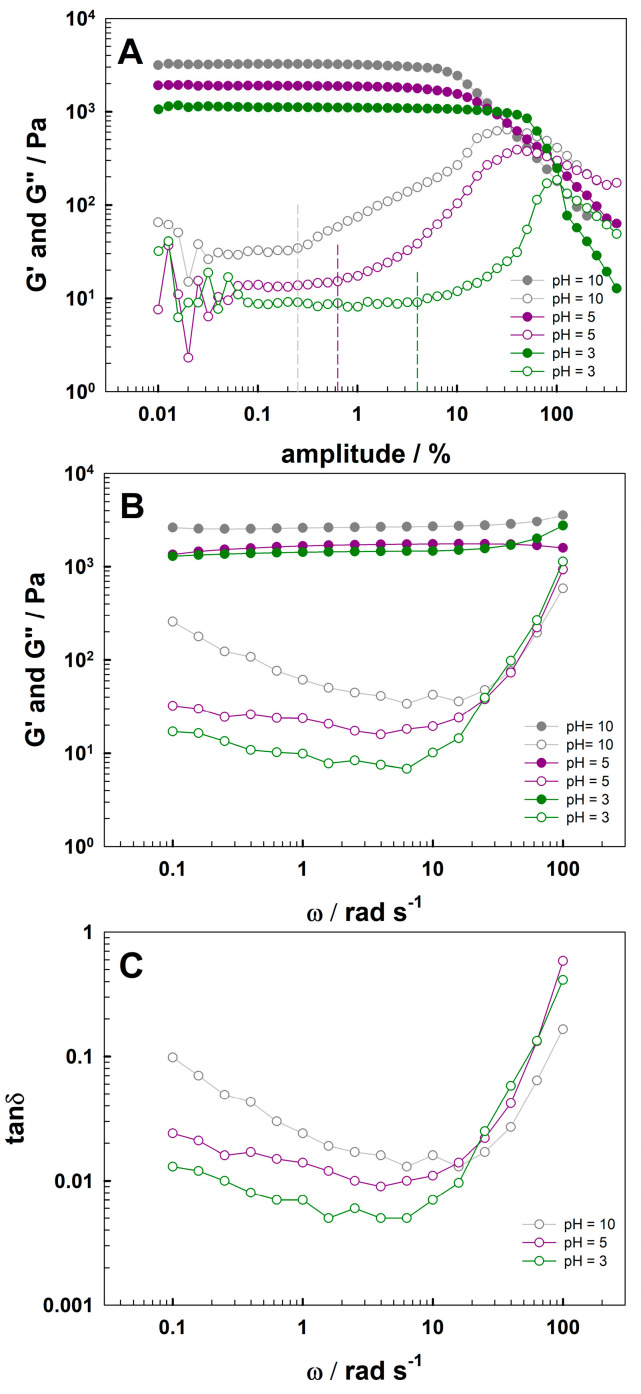
(**A**) Storage modulus G′ (solid symbols), loss modulus G″ (hollow symbols) as a function of shear strain, (**B**) storage modulus G′ (solid symbols), loss modulus G″ (hollow symbols) as a function of angular frequency, and (**C**) loss factor as a function of angular frequency—for the p(NIPA-5%APMA)-Gal-Fe gels at pH = 10 (grey), pH = 5 (purple), and pH = 3 (green).

Next, the influence of complex stoichiometry on the mechanical properties of the gels was quantitively determined by rheological measurements. Figure 6A presents the storage modulus (G′) and loss modulus (G″) as a function of the shear strain (γ) for a fixed frequency of 10 rad s^−1^. The linear viscoelastic region (LVR), where G′ and G″ are independent of the shear strain, was seen for all pH; for p(NIPA-5%APMA)-Gal-Fe at pH = 5 and 10, the LVR were narrowed. In spite of this, the storage modulus in this region was significantly higher than the loss modulus, indicating a soft solid-like state. With increasing shear strain, G′ decreased, while G″ initially increased, to further decrease after the crossover point. This behavior is called weak-strain overshoot behavior (type III behavior) [52,53]. The increase in G″ indicates the crushing of the internal structure of materials; the critical strain (deformation γ_c_ at which G″ starts to increase) increased in the order pH = 10, 5, and 3, at 0.251%, 0.634%, and 4.000%, respectively. These γ_c_ values are marked with dashed lines in Figure 6A. Decreasing critical strain could be related to increasing cross-linking density. This finding was in agreement with UV-Vis, swelling ratio and SEM investigations. For the gels, the highest crosslinking density was obtained at pH = 10, where 3:1 complexes between gallate groups and Fe(III) were formed and the value of γ_c_ was the smallest, while the lowest crosslinking density was at pH = 3, where complex stoichiometry was 1:1 and γ_c_ had the greatest value. In addition, the storage modulus increased with increasing pH, indicating that the gels became more robust. Figure 6B presents G′ and G″ at different angular frequencies, and constant amplitude γ = 0.2% chosen from the linear viscoelastic region from Figure 6A. For all the obtained hydrogels, the storage modulus was larger than the loss modulus, indicating the solid-like and elastic nature of p(NIPA-5%APMA)-Gal-Fe gels. The storage modulus changed slightly over the measured frequency range, which is typical of covalently cross-linked gels, whereas the loss modulus changed significantly. For p(NIPA-5%APMA)-Gal-Fe, G″ first decreased with increasing frequency, then increased. The nature of the metal–ligand crosslinking coordination bond has dynamic, not covalent, nature [54,55]. Nevertheless, the G′ and G″ dependence indicates that Gal-Fe are strong bonds providing additional high stability crosslinks near permanent crosslinks [56]. As was shown by Amstad et al., the relaxation time is longer for pyrogallol-modified PEG hydrogels, which are stronger than catechol analogs [57]. Figure 6C shows that the loss factors (of p(NIPA-5%APMA)-Gal-Fe) for lower frequencies were below 0.1 for all mono, bis, and tris complexes, which is typical for gel-like materials. At pH 10, the competition between ligand gallate groups and hydroxyl groups and iron(III) can take place [54]. When frequencies increase, more viscous-like behavior is predominant for all pH, with G″ approaching G′ presumably, deformation of the robust polymer structure is responsible for this response [58].

## 3. Conclusions

Gallic acid was introduced to thermoresponsive gels based on *N*-isopropylacrylamide functionalized with amino groups. This was confirmed with ^1^H NMR spectroscopy and the hydrogels were found to exhibit similar electrochemical properties as unbounded gallic acid in aqueous solution. The gallate groups attached to the polymeric network were shown to be able to create stable complexes with Fe(III) ions. The stoichiometry of the complexes so formed depended on the pH, and this was reflected in the different colors of the gels. In consequence, the temperature of the volume phase transition and the swelling ratio decreased substantially with increasing pH and changing stoichiometry from 1:1 through to 1:2 to 1:3. This creates a potential to generate a larger change in volume caused by pH. Interestingly, the volume changes exhibited by p(NIPA-5%APMA)-Gal-Fe gel were largest at a temperature close to that of the human body (~38 °C). Moreover, note that the pH sensitivity of the gels was not related to protonation or deprotonation of the acid/base groups in the polymer network, which is the typical mechanism for such environmentally sensitive hydrogel materials. Rather, in this case, changes in the stoichiometry of complexes, which act as physical crosslinking points with different functions, were responsible for pH sensitivity. The formation of complexes of different stoichiometry also strongly influenced the morphology and mechanical properties of the gel. Such modification of thermoresponsive pNIPA gel with gallic acid opens new opportunities for the development of pH- and thermosensitive gel materials.

## 4. Materials and Methods

### 4.1. Materials

*N*-isopropylacrylamide (NIPA), *N*-(3-aminopropyl)methacrylamide hydrochloride (APMA), *N*,*N′*-methylenebisacrylamide (BIS), ammonium persulfate (APS), *N*,*N*,*N′*,*N′*-tetramethylethylenediamine (TEMED), gallic acid (GA), *N*-(3-Dimethylaminopropyl)-*N′*-ethylcarbodiimide (EDC), N-hydroxysuccinimide (NHS), dimethylformamide (DMF), triethylamine (TEA), sodium nitrate (NaNO_3_), chloric acid (HCl), sodium hydroxide (NaOH), and D_2_O were purchased from Aldrich. All chemicals were used as received, except for NIPA, which was recrystallized from the toluene-hexane mixture (90:10 *v*/*v*). All solutions were prepared using high purity water obtained from a Hydrolab purification system (water conductivity: 0.05 μS·cm^−1^).

### 4.2. p(NIPA-X%APMA)-Gal Gel Preparation

p(NIPA-X%APMA)-Gal gels were synthesized by free-radical solution copolymerization. The total concentration of NIPA, APMA, and BIS was kept constant at 700 mM. BIS concentration in all samples was fixed at 1 mol.%, while the concentrations of NIPA and APMA were varied. The APMA concentration was either 2.5%, 5% and 7.5%. The pre-gel solutions were degassed and the polymerization was initiated and accelerated by APS (2 mM) and TEMED (32 mM) and carried out at 5 °C for 20 h. The gel was synthesized in bulk and in glass rods with a known diameter of 500 μm. After this time, the gel was taken from the forms and washed five times with water to remove unreacted reagents. After synthesis, the gels were transparent and colorless. In the next step, hydrogels were cut into pieces and dried. After drying, gel samples were immersed in DMF solution with NHS (25 mM) and gallic acid (20 mM) to swell. Next, TEA (25 mM) and EDC (100 mM) were added. The next day, samples were washed several times to exchange DMF for water and to remove residues. The last step involved gel modification with iron ions. For this purpose, p(NIPA-X%APMA)-Gal gels were immersed in degassed, 10 mM Fe(III) solution (pH ca. 3). Volumes of the solution were chosen to provide a 50% excess of Fe(III) to the gall groups. It was assumed that the incorporation of APMA monomer into the polymer network and its modification with gallic acid had 100% efficiency. After 10 min, the iron solution was replaced with a solution with pH = 3, 5, or 10, respectively, and gel samples were equilibrated in these solutions. To maintain ionic strength at the same level, solutions contained 1 mM NaNO_3_, except for solutions with pH = 3, (this was undertaken to avoid the influence of the ionic strength on the degree of swelling).

### 4.3. Methods

#### 4.3.1. Swelling Ratio Measurements

Modified rod-shaped gel samples were inserted into a water-jacketed cell filled with appropriate solvent. The changes in the gel volume, caused by changes in temperature, were determined directly from the changes in the sample diameter. The latter was measured using an inverted optical microscope equipped with a digital camera (Zeiss Primo Vert, Jena, Germany). During the experiments, temperature was controlled using a heated circulating bath (PolyScience, Niles, IL, USA). The swelling ratio for the rod-shaped gels can be defined as V/V_o_ = (d/d_o_)^3^, where V and V_o_ represent the equilibrium volume of the hydrogel and the initial gel volume, d denotes the diameter of the gel rod, and d_o_ is the diameter of the capillary in which the gel was synthesized. The precision of gel rod diameter measurement was better than 3%.

#### 4.3.2. Scanning Electron Microscopy

To visualize pores in the obtained gels, cross-sections of samples were analyzed using a scanning electron microscope (SEM). The SEM images were taken with a Zeiss Merlin (Zeiss, Jena, Germany) field-emission instrument. Before taking the micrographs, the hydrogel samples were dried using the lyophilization method. The samples were first frozen in liquid nitrogen to maintain their porous structure and then freeze-dried at −82 °C under 0.05 mbar pressure in a Lyophilizer Labconco FreeZone apparatus (Labconco, Kansas City, MO, USA). Before the imaging, the samples were coated with a 3 nm layer of sputtered Au–Pd alloy using a Polaron SC7620 (Quorum, Hertfordshire, UK) mini sputter coater.

#### 4.3.3. UV-Vis Spectroscopy

The spectra of complexes of modified hydrogel with iron(III) and solution of gallic acid and iron(III) in different pH were recorded using a Thermo Scientific Spectrophotometer Evolution 300 (Thermo Fisher Scientific, Waltham, MA, USA) in the range of 350–800 nm.

#### 4.3.4. Electrochemical Characterization

The cyclic voltammograms were registered in a three-electrode system, in a custom-built electrochemical cell enabling measurements to be conducted in a hydrogel without external solution. The counter and reference electrodes were placed on the bottom, as a circle and surrounding ring, the gel sample was placed on it, and the working electrode pressed onto the gel from above. The working electrode was glassy carbon, while the counter and reference electrodes were made of platinum. The hydrogels were soaked in a 0.001 M NaNO_3_ solution that served as a supporting electrolyte.

#### 4.3.5. Rheological Measurements

An Anton Paar MCR302 (Anton Paar, Graz, Austria) rheometer was used for dynamic shear rheology experiments using a set of 15 mm diameter parallel plates at a constant temperature of 20 °C. First, dynamic oscillatory strain sweep experiments were performed on the hydrogels to determine the limit of the linear viscoelastic region. The dynamic strain sweep (γ) was performed at constant frequency, ω = 10 rad·s^−1^ in the range of 0.01% to 400%. Therefore, in all the frequency sweep tests, the strain amplitude (γ) was fixed at 0.2% (within a linear viscoelastic range small enough to avoid the nonlinear response and large enough to have a reasonable signal intensity), over a frequency range of 0.01–100 rad·s^−1^. Temperature was controlled using a PolyScience circulating bath (PolyScience, Niles, IL, USA). A cap was used to keep the desired constant temperature of hydrogel samples and minimize water evaporation during rheological measurements.

#### 4.3.6. NMR Studies

^1^H NMR spectra were obtained in D2O at 22 °C using a Bruker 300 MHz spectrometer (Brucker, Billerica, MA, USA). The samples were dried at room temperature. Next the samples were ground and swollen in D_2_O.

## Data Availability

The data presented in this study are available on request from the corresponding author.
